# A contextually relevant approach to assessing health risk behavior in a rural sub-Saharan Africa setting: the Kilifi health risk behavior questionnaire

**DOI:** 10.1186/s12889-018-5710-4

**Published:** 2018-06-20

**Authors:** Derrick Ssewanyana, Anneloes van Baar, Charles R. Newton, Amina Abubakar

**Affiliations:** 1 Centre for Geographic Medicine Research Coast, Kenya Medical Research Institute (KEMRI), P.O Box 230, Kilifi, Kenya; 2 Utrecht Centre for Child and Adolescent Studies, Utrecht University, P.O Box 80140, 3508 TC Utrecht, The Netherlands; 3 Department of Public Health, Pwani University, P.O Box 195, Kilifi, Kenya; 4 Department of Psychiatry, Warneford Hospital, University of Oxford, Oxford, OX3 7JX UK

**Keywords:** Adolescents, Sub-Saharan Africa, Health risk behavior, Questionnaire, Kenya, Audio computer assisted self-interview, Validation

## Abstract

**Background:**

Health risk behavior (HRB) is of concern during adolescence. In sub-Saharan Africa, reliable, valid and culturally appropriate measures of HRB are urgently needed. This study aims at assembling and psychometrically evaluating a comprehensive questionnaire on HRB of adolescents in Kilifi County at the coast of Kenya.

**Methods:**

The Kilifi Health Risk Behavior Questionnaire (KRIBE-Q) was assembled using items on HRB identified from a systematic review and by consulting 85 young people through 11 focus group discussions and in-depth interviews with 10 key informants like teachers and employees of organizations providing various services to young people in Kilifi County. The assembled list of HRB items were back and forward translated from English to Swahili and harmonized by a panel of experts. A total of 164 adolescents completed the assembled Swahili questionnaire at baseline and two weeks later 85 of them completed the questionnaire again. A classical test theory approach was utilized for psychometric evaluation. We computed the amount of missing data at item-level to verify data quality. Scaling evaluation was assessed by spread of responses across options at an item-level. Using Gwet’s AC1 coefficient, test-retest reliability was assessed using data from the 85 adolescents who answered the questionnaire twice. Observations and completion of a brief questionnaire were done for non-psychometric evaluation of the KRIBE-Q administered via audio-computer assisted self-interview (ACASI) in Swahili language to 40 adolescents.

**Results:**

The KRIBE-Q showed high data quality, good spread of responses across options and a very good test-retest reliability (Gwet’s AC1 = 0.82). It comprised 8 components with acceptable test-retest reliability: behavior resulting in unintentional injury and violence (0.85); tobacco use (0.85); alcohol and drug use (0.96); sexual behaviors (0.94); dietary behaviors (0.60); physical activity (0.74); gambling (0.73); and hygiene behavior (0.89). About 96% of the adolescents found the ACASI private and easy to use. Prevalence of bullying (32%), physical fights (40%) and engagement in gambling (26%) was high.

**Conclusion:**

**The** KRIBE-Q assembled in this study is a psychometrically sound instrument for adolescents in rural coastal Kenya and feasible to administer via ACASI. This measure may be useful for surveys and planning interventions in similar settings.

**Electronic supplementary material:**

The online version of this article (10.1186/s12889-018-5710-4) contains supplementary material, which is available to authorized users.

## Background

Health risk behavior significantly contributes to the burden of disease and social problems among young people globally [1]. Health risk behavior (HRB) encompasses actions and related attitudes and perceptions that underlie people’s propensity to engage in activities associated with increased susceptibility to a specific disease or ill health as shown in epidemiological or social data [2, 3]. Although prioritized forms of HRB may vary across geographical and demographic contexts, some of the commonly assessed forms of HRB include: sexual behaviors resulting in unintended pregnancy and sexually transmitted diseases; alcohol, tobacco and other drug use; behavior resulting in unintentional injury or violence; unhealthy dietary behavior; poor hygiene practices; and inadequate physical activity [4–6].

HRB is particularly of concern during adolescence (10–19), mainly because this stage of development has been linked with increased impulsivity and propensity for risk taking that might result into disability and fatal outcomes [7, 8]. The leading cause of disability and mortality among adolescents are HRB. More than 3000 adolescents die every day largely from preventable causes such as road traffic accidents, falls, diarrheal diseases, iron deficiency, alcohol use, violence and self-inflicted injuries; most of which are associated with HRB. These preventable causes of mortality are also ranked among the top ten causes of disability-adjusted life-years (DALYs) among adolescents globally [1, 9]. Moreover, adolescence is a period when behaviors (either risky or protective) start or are consolidated and this has major implications for health in adulthood [9]. Governments have therefore been called upon to prioritize action and investments in prevention, in accordance with the disease and injury risk factor profiles of their adolescent populations [9].

Understanding HRB of adolescents especially from low resource settings such as sub-Saharan Africa (SSA) is partly hindered by the lack of culturally appropriate measures of HRB, considering that majority of the available HRB tools have been developed and utilized in high income settings [10]. In Kenya, which is also the setting for this current study, there is a growing body of research on adolescents’ HRB [11–17]. However, for most of these studies the authors do not explicitly inform the readership about the process of development, sources and psychometric qualities of the utilized HRB items. This status quo may imply that either: i) HRB items are often directly translated for use within the Kenyan context from a different context; or ii) that HRB researchers commonly develop new items and administer them without thoroughly ascertaining their cultural appropriateness and content validity. Although this approach of formulating new items, or using already existing ones with minimal or no modification, is relatively cheap and easy to implement; it is not often culturally informed and potentially may contribute to biased results [18]. These issues can include different forms of bias, like *item bias* which arises when items function differently or have different meaning in different contexts; *construct bias* which may arise when an instrument partially explores the domains that constitute a construct; and *method bias* that arises from differences in administration procedures or sample characteristics [18]. Another challenge for adolescent HRB research within the Kenyan context is that many studies tend to assess HRB in isolation, hence missing out important aspects such as co-occurrence of HRB of adolescents, which has for instance been explored in a few studies and found to be an important issue [19, 20]. Regional disparities within Kenya in the burden, forms and specific underlying factors for some behavior and related outcomes [21] also underscore the need for culturally appropriate and comprehensive HRB tools. An example is Kilifi County at the Kenyan coast where national level data suggests a disproportionately higher burden of sexual risk behavior outcomes e.g. a highest prevalence (21%) of teenage pregnancy [21]. The existence of such differences potentially emphasizes the presence of various context specific underlying factors [22].

There is a growing body of evidence to guide researchers on steps towards ensuring cultural appropriateness and validity of tests on health outcomes in low resources settings where still scarcity of such tests exists [18, 23, 24]. Adoption, adaptation and assembly of assessment instruments are three alternatives, but the latter two options are preferred compared to adoption since they are more likely to contribute contextually relevant tools [18]. Adoption involves translating a measure and using it in another culture or context, while adaptation involves a systematic evaluation of all aspects of an existing instrument and modifying it where needed to suit context. For assembly, a new measure is either developed directly and informed by local culture and context or alternatively items and procedures are borrowed or modified from various standardized measures [18]. Abubakar and van de Vijver [18] propose a four-step approach to adaptation and assembly of tests which involves: i) a mixed methods approach to construct clarification; ii) item development, which comprises item translation and formulation; iii) scale development, which involves refining a scale by pretesting and piloting; and iv) psychometric and non-psychometric approaches to test evaluation. We explain in depth these procedures in the methodology section of this manuscript.

This present study is conducted in Kilifi County; a rural setting at Kenyan coast, with a main objective of assembling and psychometrically evaluating a comprehensive questionnaire for assessing health risk behavior of adolescents in this environment. Owing to the sensitivity of topics in HRB, we further aimed to ensure that the psychometrically evaluated HRB questionnaire can be answered in a manner that maximizes the adolescents’ privacy.

## Methods

### Ethical consideration

The Kenya Medical Research Institute Scientific and Ethics Review Unit granted ethical approval for this study (KEMRI/SERU/CGMR-C/0047/3263). We obtained written informed consent for all participants during all recruitment processes. Consent was directly sought from participants aged 18 years and over, while for those less than 18 years, parents or legal caretakers provided consent. Permission to involve schools in this study was obtained from the Kilifi County director of education and the school head teachers. All data collected during this study was coded with non-personal identifiers to optimize privacy and confidentiality. Since HRB is a sensitive topic, the research team comprised a trained counselor who provided a health talk and also supported participants in the case of emotional distress. Information sheets on referral services (e.g. HIV/STI testing and counseling services, services to address sexual and gender based violence) were also provided to all participants during the study.

### Assembling of the questionnaire

We utilized a systematic approach recommended for adaptation of tests for sub Saharan Africa [18] to assemble and psychometrically evaluate the Kilifi Health Risk Behavior Questionnaire (KRIBE-Q) which is culturally appropriate for adolescents (10–19 years) living in Kilifi County at the Kenyan Coast. This process comprised four steps that are explained in detail in the following sections.

#### Construct clarification

A systematic review of published literature on HRB tools commonly utilized among adolescents was conducted to identify the *etic* aspects of tool assembly such as the commonly utilized HRB tools or sources of items on HRB assessment of adolescents, their psychometric properties, and the common means of HRB tool administration [10]. We also conducted 11 focus group discussions (FGDs) with 78 adolescents (10–19 years); and consultations with 7 young adults (20–30 years) and 10 local stakeholders who work extensively on adolescent issues for example clinicians, teachers, and employees of community based organizations based in Kilifi County. The FGDs lasted about 75–120 min and comprised between 7 and 9 participants. Of these, 8 sex disaggregated FGDs comprised primary and secondary school-going adolescents and took place at school. The other 3 FGDs involved adolescents living with HIV, adolescents who had dropped out of school and young adults. These 3 FGDs were conducted in a private and quiet setting. Each local stakeholder participated in an in-depth key interview (KI) lasting about 60–90 min at their convenient time and venue. The FGDs and KIs were moderated by a Research Officer either in Kiswahili or English following participants’ preference. The discussions were audio recorded and notes were taken during these sessions. The FGDs and KIs were guided by a qualitative interview and FGD guide developed to cover a wide range of adolescent HRB like alcohol, tobacco and other drug use, sexual behavior, behavior resulting in unintentional, self-harm and violence, hygiene, physical activity and dietary behaviors [25]. An open ended question concerning respondents’ perceptions about each form of behavior was asked and followed up with additional probing on specific examples or context of behavior raised by the participants. In general, they were asked to explain the specific forms of risky behaviors which they perceive as commonly undertaken by adolescents aged 10 to 19 years in Kilifi.

Through this qualitative component, we explored *emic* aspects such as forms and specific examples of prevalent HRB; local names, popular terminologies or phrases surrounding specific behavior; substances and drugs; day-to-day experiences of young people in Kilifi; and the underlying protective and risk factors for HRB [22].

#### Item development and refinement of the questionnaire

A list of items identified in the systematic review and generated from consultations during the qualitative data collection (see prior section) was prepared. The items were mainly borrowed (with some modification to suit context) from the Youth Risk Behavior Surveillance System (YRBSS) [26] which had been identified as the most commonly utilized HRB assessment tool from the systematic review [10].

These assembled set of items on HRB were translated from English to Swahili language (the national language for Kenya and lingua franca for more than 60 million people in East Africa [27]) and then back translated to English by two independent persons who were bilingual. A harmonization process of the translated version of the HRB tool was then conducted by a panel comprising two authors of this manuscript (DS and AA) and 4 research assistants from Kenya Medical Research Institute-Wellcome Trust Program (KWTRP). This panel scrutinized these items for clarity (translation and conceptual meaning) and identified specific gaps for improving the questionnaire.

Gaps were identified, mainly about aspects of HRB raised in the qualitative interviews and discussions but missing or insufficiently captured in the YRBSS. Other items were borrowed (with some modification to suit context) from the Global School –Based Student Health Survey [4], Diagnostic and Statistical Manual for Mental disorder-IV-Multiple Response-Juvenile (DSM-IV-MR-J) [28], the Pittsburgh Sleep Quality Index [29], and the international physical activity questionnaire (IPAQ-Short) [30]. The borrowed items and modifications are later described in the results section. Relevant items to capture socio-demographic variables such as sex; age; religious affiliation; and educational achievement were then added to the revised draft of the assembled items.

#### Scale development

DS and AA carefully reviewed the assembled KRIBE-Q and developed the scoring and administration procedures of the questionnaire. This step was guided by a list of questions such as, whom to survey; how to obtain permission; when (part of the year and time of the day) to collect the data; what administration procedures to follow; and how to analyze and report the findings [31]. A team of two research assistants was then trained on the administration of the KRIBE-Q. This training covered topics on: the purpose of the survey, the administration schedules; the relevance of ensuring privacy and confidentiality; and the contents of the KRIBE-Q.

#### Test evaluation

The test evaluation step consisted of both psychometric and non-psychometric evaluation of the assembled Swahili version of the KRIBE-Q. We utilized a stratified random sampling method to recruit adolescents from upper primary classes (class 5 to 8) and lower secondary classes (form 1 to 2) in a classroom setting, during a normal school day in two primary and two secondary schools representative of a peri-urban and rural setting of Kilifi County. All the schools involved in this study were situated within Kilifi Health Demography Surveillance System [31]. Prior to recruitment, upper primary and lower secondary students in these schools had been informed about the study during an afternoon session organized by a school teacher on duty. The participants were aged 10 to 19 years, and Swahili speakers. A total sample of 164 adolescents were initially administered the Swahili version of the KRIBE-Q (Time 1); and after a 14 days’ period (Time 2), 85 participants of those who took part at Time 1 were re-administered this HRB questionnaire. The assembled KRIBE-Q was administered within a classroom setting by an interviewer who read out one question after the other while the participants circled their answer options.

The psychometric evaluation of the Swahili version of the KRIBE-Q was conducted using the classical test theory [32], which involved: assessment of the data quality; scaling evaluation; and test-retest reliability, similar to steps suggested by Petrillo and colleagues [33].

The data quality and scaling evaluation utilized baseline (Time 1) item level descriptive statistics (e.g. response rates per item or question). Analysis was conducted in STATA 15 statistical package [34]. Data quality was verified by computing the amount of missing data at item-level. Acceptable data quality was achieved when no more than 5% of the data was missing per item or question [35]. Also, a greater trend of missing data towards the end of the questionnaire would likely indicate respondent fatigue, whereas greater amount of missing data by content would suggest problems with content validity [33]. Scaling evaluation (spread of responses across options) was done by assessing how appropriately the item response categories for each question/item were utilized. We considered that at least 60% of the total response categories per item should have been selected by the participants to ascertain optimal scaling evaluation.

Test-retest reliability was assessed using data of 85 adolescents who had completed the assembled Swahili version of KRIBE-Q both Time 1 and Time 2 (after a two weeks interval).

In order to calculate the test-retest reliability and prevalence of HRB, we transformed the HRB categorical variables in to binary format similar to what has been done in various HRB tool psychometric evaluation studies [36–38]. We assessed if there were statistically significant differences in HRB prevalence between Time 1 and Time 2 by finding out if there was no overlap of confidence intervals of prevalence rates of the HRB for Time 1 and Time 2 of each item.

Test-retest reliability was computed using Gwet’s AC1 coefficients [39, 40] in STATA15 software package. Gwet’s AC1 is an agreement coefficient that uses a more stable chance-adjusted index to estimate the reliability of categorical variables and has considered an improved alternative to existing inter-rater reliability statistics [41]. We used Gwet’s AC1 coefficients instead of Cohen’s Kappa coefficient [42] because of the potential “Kappa paradox” that results when low Kappa coefficient values are found despite high percentage of agreement [43]. Conversely, the Gwet’s AC1 coefficient provides more stable reliability values than Cohen’s Kappa and is less affected by prevalence and marginal probability [44]. A benchmark scale proposed by Altman [45] was used to classify Gwet’s AC1 coefficient as: ‘very good’ (0.81–1.00); ‘good’ (0.61–0.80); ‘moderate’ (0.41–0.60); ‘fair’ (0.21–0.40); and ‘poor’ (less than 0.20).

Following ascertainment of good psychometric properties of the KRIBE-Q, we wanted to ensure that this paper-and-pencil self-administered HRB questionnaire can be answered in a manner that maximizes the adolescents’ privacy. Therefore, with technical assistance from the computer programmers at the KWTRP, the KRIBE-Q was customized into an audio-computer assisted self-interview (ACASI), which is delivered via a touch-screen desktop or laptop computer. A respondent listens to the instructions and questions through a pair of headsets and selects their appropriate response option with their finger on the touch screen. We also conducted a non-psychometric evaluation of the KRIBE-Q administered via ACASI in Swahili language to a sample of 40 adolescents. The non-psychometric evaluation involved ascertainment of face validity during the interview by a research assistant who observed participants’ reaction to the ACASI. The research assistant also took note of any form of assistance required by participants during the interview and asked the participants to rank their level of privacy, interest, and easiness in completing the ACASI using a brief questionnaire.

## Results

### Characteristics of participants

The characteristics (sample size, age, and sex) of the participants that took part in the various stages of the KRIBE-Q assembly and psychometric evaluation are summarized (see Table 1).

**Table 1:** A summary of age and sex of the participants for different stages of the study

Stage of tool development	Participants (n)	Mean Age (SD)	Males (%)
Construct clarification (focus groups & key informants)	Adolescents (78)	15.0 (2.4)	53.8
	Stakeholders (10)	35.8 (2.9)	40
	Young adults (7)	24.7 (1.1)	43
Item development and refinement	Translators (2) Harmonization Panelists (6)	–	50
		–	83.3
Test evaluation			
i. Baseline (Time 1)	Adolescents (164)	14.8 (2.4)	49.4
ii. Retest (Time 2)	Adolescents (85)	14.3 (2.5)	47.1
Non Psychometric evaluation of ACASI	Adolescents (40)	14.1 (1.6)	42.5

### Construct clarification

From the systematic review, the Youth Risk Behavior Surveillance System (YRBSS) and Health Behavior in School-aged Children (HBSC) were the commonest and most comprehensive HRB assessment tools and sources of items on HRB for adolescents living with chronic conditions [10]. Based on the findings from the review [10], the YRBSS questionnaire [26], informed most of our item choices for the assembly of the KRIBE-Q. We also modified the format of the items from the YRBSS to suit the local context in Kilifi and borrowed some items from the Global School –Based Student Health Survey [4], DSM-IV-MR-J [28], the Pittsburgh Sleep Quality Index [29], and International Physical Activity Questionnaire (IPAQ-Short) [30].

Our results from the focus group discussions and the key informant interviews in Kilifi showed a number of contextually relevant forms of HRB and specific local names or jargon that we integrated in to the questionnaire. The specific modifications made included:

i) Modification of items on behavior resulting to injury and violence in order to capture injuries from motorcycles and bicycles, falls, burns and cuts; and cyber bullying (i.e. through phone text messages and social media platforms) which during the focus group discussions were found to be common among adolescents in Kilifi.
ii) Inclusion of locally relevant examples of tobacco products like shisha, ugoro, and tobacco leaves or tumbaku; examples of locally brewed forms of alcohol like mnazi, and changaa; and local names of other drugs for example bangi, ganja and makushabu (for marijuana) and miraa or mogoka (for Khat) in the questionnaire’s sections on tobacco use, alcohol and other drug use behavior.
iii) Specific locally relevant examples of healthy foods for example vegetables like kales (Sukuma wiki), amaranthus (mchicha), and potentially unhealthy locally or fatty available foods like fried chicken and viaza karai that were named by young people were also included under dietary behavior in the assembled KRIBE-Q [46].
iv) Gambling was another noteworthy form of behavior discussed by young people in Kilifi [46] which is not captured by the YRBSS questionnaire [26]. Therefore three items assessing gambling behavior were borrowed from the DSM-IV-MR-J [28], with some modifications such as giving specific examples of gambling games such as card games or ‘kamare’, lottery or scratch tickets, casino games or ‘Mchina’ and sports betting that had been mentioned in the discussions.
v) The need for assessment of quality of sleep behavior followed from engagement in social events such as parties and traditional ceremonies by adolescents, which take place in the night [22]. An item on this aspect was borrowed from the Pittsburgh Sleep Quality Index [29].
vi) Personal hygiene behavior such as poor hand washing practices, poor oral hygiene and general body cleanliness were also mentioned by young people as perceived forms of HRB in Kilifi. We therefore borrowed items (with minimal modification) on personal hygiene behavior (i.e. oral hygiene, handwashing and general body hygiene), from the Global School –Based Student Health Survey (GSHS) 2013 core questionnaire modules [4].

### Item development and refinement of the questionnaire

Suggestions from the harmonization panel were that the 5 items on safety (question 8–13) from the YRBSS [26], be replaced with the 3 items about serious injuries borrowed with some modification from the GSHS 2013 core questionnaire modules [4]. The main reason for this was because the YRBSS questions about safety majorly focused on road traffic safety (especially motor vehicle safety), whereas other forms of injury appeared to be of higher priority in the Kilifi context, many of which are captured in the GSHS questionnaire. Addition of an extra item was also proposed to capture why the identified forms of injuries occurred, and the response options reflected issues discussed by young people: for example being under the influence of alcohol or drugs, being reckless, and having no control over the incident.

Items about violence related behavior that made reference to school property in the YRBSS were modified to reflect occurrence in a general community context in order to optimally include all potential HRB.

The harmonization panel proposed that items on alcohol use behavior from the GSHS be used since they capture various problem drinking related indicators, like number of drinks consumed in a day, being in trouble or missing school due to alcohol drinking, and being intoxicated. Additionally, the GSHS items also ask about caregivers’ alcohol use unlike those in the YRSS [4, 26].

Consensus from the panel was that items on sexual orientation (heterosexual, gay and bisexual) as used in the YRBSS should be dropped from the assembled KRIBE-Q. Asking adolescents about their sexual orientation was thought as a culturally complex subject in the context of Kilifi.

There was preference for items on physical activity that assess vigorous and moderate forms of physical activity as well as sedentary lifestyle. Thus such items were borrowed from the international physical activity questionnaire (IPAQ-Short) [30].

### Scale development

An instruction manual for the administration of the KRIBE-Q outlining various procedures for observing privacy, seeking permission, scoring the items, handling data collection materials, managing data and reporting was developed. Following consultations with school authorities and the adolescents, we found that it was most convenient to administer the questionnaire at the start or mid-way into the academic term before too much academic work load and examinations. The sports’ time was suitable for this activity since this avoided interference with academic activities. Following the training, the research assistants and counselor felt prepared for data collection in the test evaluation phase.

### Test evaluation

Of the 214 adolescents who had shown interest during the recruitment stage, 76.6% (164) completed the KRIBE-Q at Time 1, and their data was basis for the data quality and scaling evaluation. Table 2 presents the participants’ characteristics.

**Table 2:** Characteristics of participants involved in the HRB tool psychometric evaluation phase

Characteristic	Time 1 (%), *N* = 164	Time 2 (%), *N* = 85
Sex		
Male	49.4	47.1
Female	50.6	52.9
Adolescent group		
Young adolescents (10–14 yrs)	41.5	50.6
Older adolescent (15–19 yrs)	58.5	49.4
School level		
Primary (Class 5–8)	45.4	57.1
Secondary (Form 1–2)	54.6	42.9
Religious affiliation		
Christian	82.2	84.7
Moslem	17.2	15.3
Other	0.6	0.0

Our results of test evaluation include 60 of a total of 69 items on health behavior from the KRIBE-Q (see Additional file 1). We excluded five multiple choice response items from the analysis; four of which were about unintentional injury and violence and one was on alcohol use. We also do not present results from four other items for which the frequency of the response options indicated that the participants had misclassified their responses. The response options of two misclassified items (use of alcohol or drugs prior to sex and use of a condom at most recent sexual intercourse) were rephrased while the other two items (attempted smoking cessation and lifetime use of prescription drugs) were dropped from the final version of the KRIBE-Q.

### Data quality and scaling evaluation

None of the items in the HRB questionnaire had 5% or more missing data which indicated that acceptable data quality was obtained. Results from scaling evaluation indicated that about 20% of the items in the questionnaire had less than 60% of their response options utilized by the study participants. Table 3 summarizes the 13 items which had less than 60% of their response options utilized and the amendments that were made to these items.

**Table 3:** A description of items with sub-optimal results from scaling evaluation and the specific amendments undertaken

Item	Utilized response options (%)	Amendments following test evaluation
13.Been threatened or injured with a weapon during the past 12 months	50.0	Retained in its original format. The participants’ responses were varied and this item is important in the context of Kilifi.
23.Attempted suicide during the past 12 months	40.0	Retained in its original format. The item is vital for assessing psychiatric and behavioral problems.
26. Smoked cigarettes during the past 30 days	42.8	Item 26 and 27 were pooled into a new item asking about “cigarette smoking or use of any other tobacco products other than cigarettes e.g. shisha, ugoro, cigas, tobacco leaves/tumbaku”
27. Used any other tobacco products during the past 30 days	28.6	
35. Has ever drank so much that they were really drunk	50.0	Retained in its original format. The item is crucial for assessing harmful alcohol use behavior
38. Ever used marijuana	40.0	Each of these items was retained in its original format since discussions showed that marijuana is common within Kilifi.
39. Had used Marijuana during the past 30 days	40.0	
40. Ever used any form of cocaine	40.0	Item 40, 42, 44, 47 were pooled into a multiple choice response item as opposed to asking separately about frequency of use of each drug.
42. Ever used heroin	40.0	
44. Ever used methamphetamines	40.0	
47. Ever used a needle to inject illegal drugs into the body	33.3	
51. Number of sexual partners during the past 3 months.	50.0	Retained in its original format. Multiple sexual partnerships were mentioned as common by young people during the construct clarification.

### Test-retest reliability

The overall Gwet’s AC1 coefficient of the assembled Swahili version of KRIBE-Q was very good (ranging from 0.81 to 0.83 across sex groups) and there were no statistically significant differences in the coefficients across sexes, adolescent age categories and level of education. Across the 8 behavior categories, the Gwet’s AC1 coefficient ranged from ‘very good’ to ‘moderate’ with majority of the behavior categories (5 out of 8 categories) having very good reliability coefficients (see Table 4).

**Table 4:** Mean Gwet’s AC1 coefficients and their 95% confidence intervals summarized by participants’ demographic and health behavior categories

Characteristic (N = 85)	Mean Gwet’s AC1	95% CI for Gwet’s AC1
Sex		
Male	0.81^a^	0.66, 0.94
Female	0.83^a^	0.70, 0.95
Adolescent group		
Young adolescents (10–14 yrs)	0.81^a^	0.67, 0.94
Older adolescent (15–19 yrs)	0.83^a^	0.69, 0.95
School level		
Primary (Class 5–8)	0.82^a^	0.69, 0.94
Secondary (Form 1–2)	0.83^a^	0.67, 0.96
Risk behavior categories		
Behavior related to Injury and Violence	0.85	0.76, 0.93
Tobacco Use behaviors	0.85	0.77, 0.94
Alcohol and other drug use behavior	0.96	0.91, 0.99
Sexual Behaviors	0.94	0.88, 0.99
Dietary Behaviors	0.60	0.43, 0.77
Physical Activity Behaviors	0.74	0.59, 0.88
Gambling behavior	0.73	0.59, 0.87
Hygiene behavior	0.89	0.82, 0.96

^a^Mean Gwet AC1 per demographic group and health behavior is based on the 60 items used in test evaluation

At an item level, Gwet’s AC1 coefficients ranged from ‘good’ to ‘very good’ (i.e. 0.63–1.00) among 83.3% of the items in the questionnaire while 11.7% had ‘moderate’ Gwet’s AC1 coefficients ranging from 0.46 to 0.59 (See Additional file 1).

### Prevalence of behavior

There were no statistically significant differences between prevalence of behavior outcomes at Time 1 and Time 2 (*n* = 85) with exception of a few items on alcohol, tobacco and drug use, dietary behavior and hygiene (See Additional file 1).

The baseline (Time 1) prevalence of unintentional injury and violence related behavior was particularly high, for example, occurrence of serious injuries during the past 12 months (61.1%), feeling unsafe in the neighborhood (14.2%), engagement in physical fights during the past 12 months (39.9%), and experience of bullying (31.7%).

Noteworthy among substance and drug use related behavior were: the early initiation (at 13 years or younger) of cigarette smoking (6%); exposure to second hand smoke (54.3%); having parents or guardians who use tobacco (18.9%); lifetime alcohol use (10.4%); recent alcohol use (5%); and the use of Khat (14%).

From Time 1 data, about 11% of the adolescents were sexually active and 5.5% of the total respondents (representing about 45% of the sexually active) had their first sexual intercourse at the age of 13 years or less. Also 6.1% (equivalent to 43% of the sexually active) did not use any method for prevention of pregnancy during their most recent sexual intercourse.

Other important behavior outcomes were that close to 23% of the adolescents did not engage in vigorous or moderate physical activity within the past 7 days; and almost a quarter (24%) spent 3 or more hours engaging in sedentary activities on a typical day. Engagement in gambling behavior within the past 12 months was common (26%) and so was the lack of adequate handwashing with soap after using a latrine /toilet use among 28.8% of the adolescents.

### Non-psychometric evaluation of the KRIBE-Q delivered via ACASI

We collected data on user experience of the customized audio-computer assisted self-interview (ACASI) from 40 adolescents of mean age 14.1 years (SD = 1.62), of whom 57.5% were female and 60% were young adolescents (10–14 years). Majority (95%) of the adolescents rated the interview (ACASI) as either ‘very easy’ or ‘just okay’ or 90% found it as either ‘very interesting’ or ‘a bit interesting’. Almost all (97.5%) of the adolescents ‘strongly agreed’ or ‘agreed’ that the interview was confidential and that they were comfortable during the ACASI exercise. From a test administrator’s observation, he either ‘agreed’ or ‘strongly agreed’ that 97.5% of the participants seemed comfortable and confident while using the ACASI. Majority (82.5%) did not require any assistance at all when using the ACASI while the rest needed minimal assistance especially in reminding them how to maneuver to the next item on the computer.

## Discussion

Our findings from construct clarification show that adolescents in rural coastal Kenya have either experienced or are familiar with most of the constructs of HRB utilized in measures like YRBSS and GSHS which were developed in other settings. Although this demonstrates good conceptual equivalence, our experience was that in-depth consultations with the adolescents and key informants, combined with a harmonization process are vital procedures especially for identifying priority forms of behavior to focus the HRB tool development and capturing common semantics and idiomatic aspects of HRB utilized by adolescents in the study setting. We also found the harmonization process as fundamental in refining the borrowed items especially in connection to cultural appropriateness and inclusiveness of relevant behavior aspects voiced by the adolescents.

The finding that none of the items had 5% or more missing data suggests that construct clarification and item refinement significantly improved clarity and suitability of the HRB items assembled for the adolescents in the study setting. Moreover, this was further demonstrated by the fact that both young and older adolescents capably completed the assembled HRB questionnaire. The completeness of the items also potentially indicates the absence of response fatigue. However, we found that four of the sixty nine assembled items had some misclassified responses; a problem we attribute to ambiguity of the response options. For example, one of the items which asked a participant whether he or she used a condom during the last sexual intercourse initially had the following response options: “I have never had sexual intercourse”, “Yes”, “No” and we recognized a tendency for participants to select the answer option “No” while they actually meant “I have never had sexual intercourse”. For this item, we finally modified the response options as follows: “A. I have never had sexual intercourse”, “B. Yes, I used a condom”, “C. No, I had sexual intercourse but did not use a condom” so as to reduce the ambiguity. In line with our refinement of the misclassified items, there has been evidence showing that more extensive verbal labeling of response options is associated with higher reliability [47].

The majority of the assembled HRB items demonstrate good spread of responses across options however about 20% of the assembled HRB items had sub-optimal scaling evaluation. We think this may have been due to very low prevalence of certain forms of behavior such as drug use and suicidal behavior in this setting, which meant that certain response options were often redundant. Indeed the number of response categories for all these items were within the recommended ranges of four to nine options [48]. Rattray and colleagues [49] advise that although redundant response options may warrant deletion of an item, it is always crucial to refer to the original research question and retain items that are thought to reflect important underlying theoretical domains. We therefore addressed such scaling problems by for example pooling items on rare forms of behavior into a single item as opposed to asking about the frequency of each single behavior. As an example, instead of asking participants how many times in their lives they had used cocaine, heroin, methamphetamines and steroid pills without a doctor’s prescription separately, we pooled them into a single multiple choice objective item asking “During your life, have you ever used the following drugs: A. Cocaine, B. Heroin, C. Methamphetamines, D. Steroid pills without a doctor’s prescription”.

Our findings support acceptable test-retest reliability of the majority of the assembled items of the KRIBE-Q for the adolescents in the rural coastal Kenyan setting. There was acceptable reliability irrespective of sex, age category and education level as shown by the overlapping confidence intervals of overall mean Gwet’s AC1 coefficient across these groups in Table 4. Overall, behavior categories of alcohol and other drug use, sexual activity, tobacco use and dietary behavior demonstrated higher test-retest reliability than the dietary behavior and physical activity categories. These findings are consistent with those reported in a study on reliability of the Youth Risk Behavior Survey questionnaire among high school students in the district of Columbia, USA [6]. One explanation for the low reliability of dietary behavior items may be that adolescents’ diet frequently changes over a short period of time which makes it difficult to reliably recall behaviors related to their nutrition. Another explanation offered by Brener and colleagues is that behaviors surrounding substance use and sexual activity are likely to be more salient to adolescents and thus more reliably recalled compared to dietary behavior or physical activity [6].

Although test-retest reliability was high, we suspect that due to very low prevalence of specific forms of behavior, this potentially resulted to none overlap of confidence intervals (statistically significance of differences) of prevalence rates of some behavior on first (Time 1) and second test (Time 2) especially for items assessing alcohol and drug use behavior. However, the inconsistence in prevalence may also suggest response errors for example arising from recall bias, social desirability bias or there may be actual changes in prevalence. Thus the results on prevalence of the behavior in our study should be treated with caution and may need much larger sample sizes to determine if there are actual differences.

Our findings indicate that adolescents frequently report occurrence of behavior resulting to intentional and unintentional injuries like bullying and physical fights, however such results need to be replicated in larger quantitative studies in this specific study setting. These findings are however not surprising since other larger studies also report a high occurrence of bullying (between 58 and 82%), conduct problems and involvement in physical fights among Kenyan adolescent students [50, 51]. High occurrence of early sexual debut and unprotected sex among our sexually active study participants further highlights the need for more sexual and reproductive health research and interventions in rural coastal Kenya; where adolescent sexual health is reported as still poor [21, 22]. Noteworthy, adolescents also endorsed other infrequently researched forms of behavior in the context of rural coastal Kenya such as gambling behavior, poor personal hygiene and physical inactivity. This potentially points to the need for more investment in adolescent health research in this setting.

This study’s findings from non-psychometric evaluation of the HRB questionnaire delivered via ACASI highlight the feasibility of using ACASI for collecting sensitive data such as information on HRB among adolescents in this rural low resource setting. In support of our findings, ACASI has previously been used among the adolescent population in Kilifi [52], as well as among an older age-group within the Kenyan setting [53, 54]. Overall, these studies have found the ACASI to improve quality of response to sensitive questions, to decrease socially desirable responses, and has also suitably been used for collecting data among participants with low formal education [53, 54].

As a major strength of our study is seen that we utilized a systematic approach to tool assembly and psychometric evaluation recommended for low resource settings in SSA [18] to develop a comprehensive culturally appropriate measure of HRB for this setting. Nonetheless, one of our study limitations is that we assembled and psychometrically evaluated the KRIBE-Q using a school attending adolescent sample and yet between 83 and 91% of Kenyan adolescents are enrolled in primary and lower secondary [55, 56]. Although this was the case, we believe that we satisfactorily captured important HRB constructs and examples from other young people like adolescents who dropped out of school, young adults, and adolescents living with chronic illnesses during the construct clarification phase. Also given the nature of items and their scoring procedure, it was not possible to perform traditional analysis for construct validity such as factorial analysis. However, this study ascertained the face and content validity as well as reliability of the KRIBE-Q. Future studies may explore predictive or criterion validity of this tool. Another limitation stems from the self-reported nature of HRB assessment utilized in our study as self-reports on sensitive topics are at times associated with self-desirability and recall bias [57]. Self-reports are however universally applied methods for HRB assessment and in our current study we strictly observed confidentiality to counter bias associated with self-reported behavior. Lastly, some of the results from this study, for example on prevalence of HRB, need replication utilizing a larger and more diverse adolescent sample.

## Conclusions

The assembled Swahili version of the KRIBE-Q in this study can be considered as reliable and its content is valid for assessing HRB of adolescents in a rural coastal Kenyan setting. Moreover, its mode of administration via ACASI was characterized as easy to answer; private and comfortable; and an enjoyable experience by the adolescents in this setting. This assembled KRIBE-Q comprises 8 components namely: behavior related to unintentional injury and violence; tobacco use behaviors; alcohol and other drug use behavior; sexual behaviors; dietary behaviors; physical activity behaviors; gambling behavior; and hygiene behavior.

We took a systematic mixed method approach for adaptation of tests for resource poor settings. We therefore expect that this assembled questionnaire will be useful in surveys evaluating adolescents’ lifestyle; and in planning and evaluating interventions aimed at addressing adolescent health, especially in resource poor settings at the Kenyan coast where such programs are currently scarce.

## Additional file

Additional file 1: A summary of the scaling evaluation, test-retest reliability and behavioral prevalence at baseline (Time 1) and retest (Time 2) for the items in the Kilifi Health Risk Behavior Questionnaire (KRIBE-Q). The data summarizes the utilization of response categories (reported in percentage); the test-retest reliability (represented by Gwet’s AC1 coefficient); overall prevalence of behavior at baseline (Time1) (reported in percentage); and prevalence of behavior (reported in percentage) for the sub-sample (85) that completed the questionnaire at both phases. The summarized data comprises 60 out of the total 69 items on health behavior in the KRIBE-Q. (XLSX 14 kb)

## Abbreviations

ACASI: Audio-Computer Assisted Self-Interview
DALYS: Disability Adjusted Life Years
DSM-IV-MR-J: Diagnostic and Statistical Manual for Mental disorder-IV-Multiple Response-Juvenile
GSHS: Global School –Based Student Health Survey
HBSC: Health Behavior in School-aged Children
HIV: Human Immunodeficiency Virus
HRB: Health Risk Behavior
IPAQ: International physical activity questionnaire
KRIBE-Q: Kilifi Health Risk Behavior Questionnaire
KWTRP: Kenya Medical Research Institute-Wellcome Trust Program
SD: Standard Deviation
SSA: Sub-Saharan Africa
STI: Sexually Transmitted Infection
YRBSS: Youth Risk Behavior Surveillance System

## References

[1] Gore FM, Bloem PJ, Patton GC, Ferguson J, Joseph V, Coffey C, Sawyer SM, Mathers CD (2011). Global burden of disease in young people aged 10–24 years: a systematic analysis. Lancet.

[2] DiClemente RJ, Hansen WB, Ponton LE (2013). Handbook of adolescent health risk behavior.

[3] Lupton D. Health Risk Behavior. In: Ritzer G, editor. Blackwell Encyclopedia of Sociology. New Jersey: Blackwell Publishing; 2007.

[4] Global School-Based Student Health Survey (GSHS). 2013 Core questionnaire module. http://www.who.int/chp/gshs/GSHS_Core_Modules_2013_English.pdf?ua=1. Accessed 18 Dec 2017.

[5] Roberts C, Currie C, Samdal O, Currie D, Smith R, Maes L (2007). Measuring the health and health behaviours of adolescents through cross-national survey research: recent developments in the health behaviour in school-aged children (HBSC) study. J Public Health.

[6] Brener ND, Collins JL, Kann L, Warren CW, Williams BI (1995). Reliability of the youth risk behavior survey questionnaire. Am J Epidemiol.

[7] Steinberg L (2008). A social neuroscience perspective on adolescent risk-taking. Dev Rev.

[8] Galvan A, Hare T, Voss H, Glover G, Casey B (2007). Risk-taking and the adolescent brain: who is at risk?. Dev Sci.

[9] WHO. Global Accelerated Action for the Health of Adolescents (AA-HA!): guidance to support country implementation. Summary. Geneva: World Health Organization; 2017.

[10] Ssewanyana D, Nyongesa MK, Baar A, Newton CR, Abubakar A (2017). Health risk behavior among chronically ill adolescents: a systematic review of assessment tools. Child Adolesc. Ment. Health.

[11] Puffer ES, Meade CS, Drabkin AS, Broverman SA, Ogwang-Odhiambo RA, Sikkema KJ (2011). Individual-and family-level psychosocial correlates of HIV risk behavior among youth in rural Kenya. AIDS Behav.

[12] Embleton L, Nyandat J, Ayuku D, Sang E, Kamanda A, Ayaya S, Nyandiko W, Gisore P, Vreeman R, Atwoli L (2017). Sexual behavior among orphaned adolescents in western Kenya: a comparison of institutional-and family-based care settings. J Adolesc Health.

[13] Onywera VO, Muthuri SK, Hayker S, Wachira L-JM, Kyallo F, Mang’eni RO, Bukhala P, Mireri C (2016). Results from Kenya’s 2016 report card on physical activity for children and youth. J Phys Act Health.

[14] Otieno A, Ofulla A. Drug abuse in Kisumu town western Kenya. Afr J Food Agric Nutr Dev. 2009;9(3):

[15] Ndetei DM, Khasakhala LI, Mutiso V, Ongecha-Owuor FA, Kokonya DA (2010). Drug use in a rural secondary school in Kenya. Subst Abus.

[16] Ruto SJ (2009). Sexual abuse of school age children: evidence from Kenya. J Int Cooperation Educ.

[17] Juma M, Askew I, Alaii J, Bartholomew LK, van den Borne B (2014). Cultural practices and sexual risk behaviour among adolescent orphans and non-orphans: a qualitative study on perceptions from a community in western Kenya. BMC Public Health.

[18] Abubakar A, van de Vijver FJ. How to adapt tests for sub-Saharan Africa. In: Handbook of Applied Developmental Science in Sub-Saharan Africa. Eedn. New York: Springer; 2017. p. 197–212.

[19] Brener ND, Collins JL (1998). Co-occurrence of health-risk behaviors among adolescents in the United States. J Adolesc Health.

[20] Nelson MC, Gordon-Larsen P (2006). Physical activity and sedentary behavior patterns are associated with selected adolescent health risk behaviors. Pediatrics.

[21] Kenya National Bureau of of Statistics (KNBS) (2014). 2015 Kenya Demographic and Health Survey, Key Indicators.

[22] Ssewanyana D, Mwangala PN, Marsh V, Jao I, van Baar A, Newton CR, Abubakar A (2017). Young people’s and stakeholders’ perspectives of adolescent sexual risk behavior in Kilifi County, Kenya: a qualitative study. J Health Psychol.

[23] Holding P, Abubakar A, P. W: Where there are no tests: a systematic approach to test adaptation. In: Cognitive Impairment: causes, diagnosis and treatment. Volume 189–200, edn. Edited by Landow ML. New York: Nova Science Publishers; 2009.

[24] Harachi TW, Choi Y, Abbott RD, Catalano RF, Bliesner SL (2006). Examining equivalence of concepts and measures in diverse samples. Prev Sci.

[25] World Health Organization (WHO). 2015 Global school-based student health survey (GSHS) purpose and methodology. http://www.who.int/chp/gshs/methodology/en/ (Accessed 22 May 2018).

[26] Centers for Disease Control and Prevention (CDC). 2015 State and Local Youth Risk Behavior Survey. https://ftp.cdc.gov/pub/data/YRBS/2015/2015_hs_questionnaire.pdf. Accessed 20 May 2018.

[27] UCLA Language Materials Project. Swahili http://www.lmp.ucla.edu/Profile.aspx?menu=004&LangID=17. Accessed 17 Dec 2017.

[28] Fisher S (2000). Developing the DSM-IV-DSM-IV criteria to identify adolescent problem gambling in non-clinical populations. J Gambl Stud.

[29] Buysse DJ, Reynolds CF, Monk TH, Berman SR, Kupfer DJ (1989). The Pittsburgh sleep quality index: a new instrument for psychiatric practice and research. Psychiatry Res.

[30] Craig CL, Marshall AL, Sjöström M, Bauman AE, Booth ML, Ainsworth BE, Pratt M, Ekelund U, Yngve A, Sallis JF (2003). International physical activity questionnaire: 12-country reliability and validity. Med Sci Sports Exerc.

[31] Centers for Disease Control and Prevention (CDC). A guide to conducting your own youth risk behavior survey. Atlanta: Center for Disease Control and Prevention; 2014.

[32] Crocker L, Algina J. Introduction to classical and modern test theory. California: ERIC; 1986.

[33] Petrillo J, Cano SJ, McLeod LD, Coon CD (2015). Using classical test theory, item response theory, and Rasch measurement theory to evaluate patient-reported outcome measures: a comparison of worked examples. Value Health.

[34] StataCorp L (2014). Stata 13.

[35] Schafer JL (1999). Multiple imputation: a primer. Stat Methods Med Res.

[36] Brener ND, Kann L, McManus T, Kinchen SA, Sundberg EC, Ross JG (2002). Reliability of the 1999 youth risk behavior survey questionnaire. J Adolesc Health.

[37] Baheiraei A, Hamzehgardeshi Z, Mohammadi M, Nedjat S, Mohammadi E (2012). Psychometric properties of the Persian version of the youth risk behavior survey questionnaire. Iran Red Crescent Med J.

[38] Guedes DP, Lopes CC (2010). Validation of the Brazilian version of the 2007 youth risk behavior survey. Rev Saude Publica.

[39] Gwet KL (2014). Handbook of inter-rater reliability: the definitive guide to measuring the extent of agreement among raters: advanced analytics, LLC.

[40] Gwet K (2002). Kappa statistic is not satisfactory for assessing the extent of agreement between raters. Stat Methods Inter-rater Reliability Assess.

[41] Gwet KL (2008). Computing inter-rater reliability and its variance in the presence of high agreement. Br J Math Stat Psychol.

[42] Cohen J (1968). Weighted kappa: nominal scale agreement provision for scaled disagreement or partial credit. Psychol Bull.

[43] Cicchetti DV, Feinstein AR (1990). High agreement but low kappa: II. Resolving the paradoxes. J Clin Epidemiol.

[44] Wongpakaran N, Wongpakaran T, Wedding D, Gwet KL (2013). A comparison of Cohen’s kappa and Gwet’s AC1 when calculating inter-rater reliability coefficients: a study conducted with personality disorder samples. BMC Med Res Methodol.

[45] Bland D, Altman D (1991). Practical statistics for medical research.

[46] Ssewanyana D, Abubakar A, van Baar A, Mwangala PN, Newton CR (2018). Perspectives on underlying factors for unhealthy diet and sedentary lifestyle of adolescents at a Kenyan coastal setting. Front Public Health.

[47] Alwin DF, Krosnick JA (1991). The reliability of survey attitude measurement: the influence of question and respondent attributes. Sociol Methods Res.

[48] Alwin DF. Information transmission in the survey interview: number of response categories and the reliability of attitude measurement. Sociol Methodol. 1992:83–118.

[49] Rattray J, Jones MC (2007). Essential elements of questionnaire design and development. J Clin Nurs.

[50] Brown DW, Riley L, Butchart A, Kann L (2008). Bullying among youth from eight African countries and associations with adverse health behaviors. Pediatr Health.

[51] Ndetei DM, Ongecha FA, Khasakhala L, Syanda J, Mutiso V, Othieno CJ, Odhiambo G, Kokonya DA (2007). Bullying in public secondary schools in Nairobi, Kenya. Child Adolesc Ment Health.

[52] Otiende M, Abubakar A, Mochamah G, Walumbe D, Nyundo C, Doyle AM, Ross DA, Newton CR, Bauni E (2017). Psychometric evaluation of the major depression inventory among young people living in coastal Kenya. Wellcome Open Res.

[53] Waruru AK, Nduati R, Tylleskär T (2005). Audio computer-assisted self-interviewing (ACASI) may avert socially desirable responses about infant feeding in the context of HIV. BMC Med Inform Decis Mak.

[54] Van Der Elst EM, Okuku HS, Nakamya P, Muhaari A, Davies A, McClelland RS, Price MA, Smith AD, Graham SM, Sanders EJ (2009). Is audio computer-assisted self-interview (ACASI) useful in risk behaviour assessment of female and male sex workers, Mombasa, Kenya?. PLoS One.

[55] The United Nations Children's Fund (UNICEF) Kenya. Statistics https://www.unicef.org/infobycountry/kenya_statistics.html. Accessed 18 June 2018.

[56] UNESCO: Education in Kenya. Progress towards the six Education for all goals in Kenya, Sub-saharan African and the world. https://en.unesco.org/gem-report/sites/gem-report/files/EDUCATION_IN_KENYA_A_FACT_SHEET.pdf. Accessed 22 Jan 2018.

[57] Krumpal I (2013). Determinants of social desirability bias in sensitive surveys: a literature review. Qual Quant.

